# Hématome sous-capsulaire du foie rompu compliquant une stéatose hépatique aiguë gravidique

**DOI:** 10.11604/pamj.2014.19.38.4009

**Published:** 2014-09-16

**Authors:** Mouhssine Doumiri, Marie Elombila, Nezha Oudghiri, Anas Tazi Saoud

**Affiliations:** 1 Service d'Anesthésie –Réanimation, Hôpital Maternité Souissi, Centre Hospitalier Universitaire, Rabat, Maroc

**Keywords:** Hématome sous-capsulaire, foie rompu, complication rare de la grossesse, subcapsular hematoma, Ruptured liver, rare complication of pregnancy

## Abstract

L'hématome sous-capsulaire du foie est une complication rare de la grossesse, survenant le plus souvent dans le cadre d'une pré éclampsie ou d'un HELLP syndrome (Hemolysis, Elevated Liver enzymes, and Low Platelets Syndrome). Rare sont les cas décris au cours d'une stéatose hépatique aiguë gravidique. Nous rapportons le cas d'une parturiente de 33 ans, multipare, sans antécédents, admise aux urgences au terme d'une grossesse à 37 semaines d'aménorrhées, pour pré éclampsie compliquée d'une stéatose hépatique aiguë gravidique. L’échographie hépatique réalisée à l'admission était sans anomalie. Une césarienne a été réalisée en urgence devant une souffrance fœtale aiguë, au cours de laquelle a été mise en évidence une rupture de l'hématome sous capsulaire du foie s'accompagnant d'un état de choc hémorragique. La prise en charge a consisté à une polytransfusion et packing perihépatique. Le retrait du packing n'a été réalisé qu'au quatrième jour, après stabilisation clinicobiologique et régression de l'encéphalopathie hépatique. L’évolution en réanimation a été favorable avec sortie de la patiente au vingtième jour. La rupture de l'hématome sous-capsulaire du foie est extrêmement dangereuse et à haut risque materno-fœtal. L'association à la stéatose hépatique aggrave le pronostic.

## Introduction

L'hématome sous-capsulaire du foie est une complication rare de la grossesse. Son incidence varie entre 1/45 000 et 1/225 000 naissances [1, 2]. Il survient dans le cadre d'une pré-éclampsie, d'un HELLP (Hemolysis, Elevated Liver enzymes, and Low Platelets) Syndrome ou rarement d'une stéatose hépatique aiguë gravidiques [3, 4]. La rupture capsulaire hépatique est associée à une mortalité maternelle et fœtale importantes. Elle est souvent découverte lorsque le tableau clinique se complique d'un choc hémorragique. Son association avec la stéatose hépatique aiguë gravidiques complique la prise en charge et aggrave plus le pronostic materno-fœtal. Sa prise en charge est multidisciplinaire et conservatrice le plus possible.

## Patient et observation

Madame N, âgée de 33 ans, III geste, III pare, enceinte de 37 semaines d'aménorrhées, sans antécédent, a été admise aux urgences, pour céphalées, épigastralgies, nausées et vomissements. À l'examen clinique: patiente consciente, apyrétique, se présentait avec un ictère, une pression artérielle élevée (175/110 mmHg) accompagnée d'une protéinurie à + + + au labstix, une hauteur utérine à 30 cm, sans contraction utérine, col long fermé postérieur, poche des eaux intact et présentation céphalique, l'enregistrement du rythme cardiaque fœtal (RCF) était sans anomalie. L’échographie obstétricale objectivait une grossesse mono fœtale évolutive, liquide amniotique normal et absence de retard de croissance intra-utérin. L’échographie hépatique était normale. La biologie retrouvait des enzymes hépatiques élevées (ASAT: 855 UI/L, ALAT: 582 UI/L), bilirubine conjuguée à 85 mg/l, bilirubine indirect à 10 mg/l). L'hémogramme objectivait un taux de plaquettes à 65 000 éléments /mm^3^, une hyperleucocytose à 18000/mm^3^ avec une hémoglobine à 10,4 g /dl. Le taux de prothrombine était à 55%. INR à 1,5. La créatinémie à 15 mg / l, l'urée sanguine à 0,9 mmol/l associé à une hypoglycémie à 0, 5 g/L. Les sérologies hépatiques A, B, et C ont été demandées et se révéleront négatives. Le diagnostic d'une complication hépatique grave de la prééclampsie (stéatose hépatique aigue gravidique) a été posé selon le contexte clinico-biologique. L'hypertension artérielle systolique a été jugulée par deux bolus de 0,5mg de nicardipine, l'hypoglycémie a été traitée par une perfusion du Sérum glucosé à 10%.

**Figure 1:**
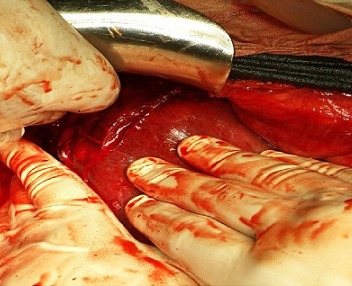
Exploration du foie après extraction fœtale au cour d’une césarienne montre un hématome sous capsulaire du foie droit rompu

La patiente a présenté une heure après son admission une souffrance fœtale aiguë sur l'enregistrement du rythme cardiaque fœtal: décélérations sévère jusqu’ a 60 BPM et prolongées. Une césarienne a été réalisée en urgence en choisissant l'anesthésie générale, devant la présence d'une coagulopathie et des décélérations sévères, avec extraction d'un nouveau né d'apgar 3:10 à 1min qui est passé à 5: 10 à 5min, puis intubé et ventilé mais décédé après 6 heures. À la fin de l'hystérorraphie, une hypotension maternelle à 70/30 mmHg a été survenue avec hemopéritoine abondant estimé à 1500 ml, obligeant un élargissement de la laparotomie en médiane, pour exploration de l'origine du saignement, mettant en évidence un hématome sous-capsulaire du lobe hépatique droit rompu et étendu jusqu’ au lobe gauche (Figure 1).

Devant cet état de choc hémorragique, la prise en charge avait consisté en un remplissage vasculaire par 1000 ml de sérum salé à 9‰, Monitorage invasif de la pression artérielle, une transfusion de 06 culots globulaires et de 06 plasmas frais congelés. L'acide tranexamique (Exacyl^®^) était utilisé avec 1g en 10 min suivie par 1g en perfusion sur 8h. Ocytocine à 40 UI en perfusion de 30min, Méthérgin à 0,2 mg en intramusculaire et misoprostol à 1000 µg par voie rectale ont été donnés pour avoir une rétraction utérine satisfaisante. Un packing périhépatique a été réalisé dés la découverte de hématome pour arrêter le saignement. Ces mesures ont permis d'obtenir un état hémodynamique stable et une coagulation satisfaisante (INR à 1,2, TP à 60% et PLQ à 70000/ml) une hémoglobine à 10g/dl en fin d'intervention. La patiente a été transférée en réanimation intubée, ventilée et sédatée.

Au deuxième jourpostopératoire, s'est installé une encéphalopathie hépatique stade III avec une aggravation de l'insuffisance rénale (créatinémie à 29 mg/l), de la coagulopathie, de l'hyponatrémie (120 mmol/l) et apparition d’œdème pulmonaire. La gestion de l'encéphalopathie hépatique a consisté à un control de l'hypertension intracrânien par mannitol à 10% (150ml toutes les 6 heures) et sérum sale hypertonique à 3% (100ml), une reprise de la diurèse à 50 ml/heure sous traitement diurétique, une correction de l'hyponatrémie et de l'hypoglycémie, une transfusion par 04 culots globulaires et de 06 plasmas frais congelés pour avoir un INR à 1,3, un TP à 54%, des plaquettes à 720000, un fibrinogène à 1,5 g/l et un hémoglobine à 10g/dl et une antibiothérapie à base de céphalosporine 3 génération et ciprofloxacine pendant 10 jour devant une pneumopathie bactérienne. Le packing n'a pu être retiré qu'au quatrième jour après stabilisation de la patiente et sans incident ou recours à la transfusion. Une biopsie hépatique était nécessaire pour confirmer la stéatose hépatique mais devant l’état hépatique et risque de saignement, cette biopsie à été abandonnée. Evolution était marquée par une régression de l'encéphalopathie hépatique, une amélioration de la fonction rénale et extubation au dixième jour. La patiente a quitté l'hôpital au vingtième jour.

## Discussion

Des cas d’ HSCF ont été rapportés dans la littérature chez la femme enceinte surtout avec un HELLP syndrome et ont été associés à une mortalité maternelle estimée à 50 à 75% et une mortalité fœtale à 60 à 80 [5]. Alors que la stéatose hépatique constitue une cause exceptionnelle [4]. Le diagnostic clinique de l'HSCF doit être évoqué devant une douleur de l'hypochondre droit, nausée et vomissements [6] ou ictère dans un contexte d'HTA. Devant ces signes une échographie hépatique est systématique. L'hématome hépatique parait sous forme d'image hétérogène hypoéchogène par rapport au reste du parenchyme. L'association avec un épanchement intrapéritonéal fait suspecter une fissure voir une rupture de l'hématome [7]. L’échographie hépatique permet en outre le suivi de l'hématome non rompue si abstention chirurgicale.

Dans notre cas la souffrance fœtale a révèle une hémorragie du foie et l’échographie hépatique n'a pas était performante pour posé le diagnostic et préparer la patiente par correction de sa coagulopathie et avoir une stratégie opératoire (type d'incision, paking prêt, envisager une embolisation). La TDM ou l'imagerie par résonance magnétique, plus performantes dans l'exploration hépatique, sont peu utilisées en pratique. L'angiographie hépatique est rarement envisageable en urgence [7]. Dans le contexte d'une rupture de HSCF une césarienne doit être faite en urgence pour extraction fœtale et une mise en place d′un packing du foie [3]. Ce dernier permet une survie maternelle, à 80% [1]. Alors l'embolisation hépatique sélective est aussi efficace avec un taux de survie maternelle à 90% [1].

Les autres techniques comme la ligature chirurgicale des artères hépatiques ou la résection des plages de nécrose hépatique sont associées à une mortalité maternelle importante supérieure à 30% [1]. La réanimation periopératoire repose sur la gestion du choc hémorragique (remplissage par 1 litre de cristalloïdes, monitorage invasif de la pression artérielle, tolérer une hypotension artérielle avant l'hémostase: PAS à 80-90 mmgHg et PAM à 65 mmHg, monitorage de la coagulation par des tests de routine comme TP, TCA, Fibrinogène, INR, D-dimères, PDF, ou par thromboélastométrie si disponible pour guider le traitement de la coagulopathie du choc hémorragique, l'apport de la transfusion sanguine doit être précoce par les culots globulaires, le plasma frais congelé et les plaquettes si sont inférieures à 50000/mm^3^ en respectant un rapport de 1/1/1, fibrinogène, cryoprécipité, facteur VII activé, corriger l’ acidose métabolique, l'hypocalcémie liée à la transfusion massive, corriger l'hypothermie et utiliser les anti-fibrinolytiques comme l'acide tranexamique) [8]. La ventilation mécanique doit être réalisée avec un volume courant bas de 6 ml/kg avec objectif de PaCO2 entre 37- 41mmHg. Dans le postopératoire la gestion de l'encéphalopathie est basée sur la ventilation mécanique, traitement de HTIC par osmothérapie: mannitol et serum salé hypertonique, sédation par benzodiazépines ou propofol voir barbituriques, une normalisation de la natrémie et de la glycémie, une correction de la coagulation, les diurétiques pour avoir une diurèse conservé si non une épuration extrarénale ( hémodialyse continue), une prévention de l'hémorragie digestive et une antibiothérapie en cas d'infections [9]. En cas de HSCF non rompu, l'extraction du bébé s'impose, après correction de la coagulopathie, pour stopper l’évolution des lésions hépatiques liées à la pré-éclampsie et la stéatose hépatique aiguë gravidique. En postopératoire, l'hématome est surveillé étroitement afin de diagnostiquer toute fissure ou rupture.

## Conclusion

La stéatose hépatique aiguë gravidique est une complication grave de la pré éclampsie. La rupture de l'HSCF est à haut risque de décès materno-foetal. L'association à la stéatose hépatique aggrave le pronostic. La survie dépend de la précocité de la PEC, dont l'approche doit être agressive et multidisciplinaire.
